# Knowledge Claims, Jurisdictional Control and Professional Status: The Case of Nurse Prescribing

**DOI:** 10.1371/journal.pone.0077279

**Published:** 2013-10-04

**Authors:** Marieke Kroezen, Liset van Dijk, Peter P. Groenewegen, Anneke L. Francke

**Affiliations:** 1 Netherlands institute for health services Research (NIVEL), Utrecht, The Netherlands; 2 Department of Sociology and Department of Human Geography, Utrecht University, Utrecht, The Netherlands; 3 Department of Public and Occupational Health, EMGO Institute for Health and Care Research (EMGO+), VU University Medical Center, Amsterdam, The Netherlands; Edinburgh University, United Kingdom

## Abstract

Over the past decades, professional boundaries in health care have come under pressure, and the expansion of prescriptive authority to include nurses touches on issues of professional domains and interprofessional competition. Knowledge claims play an important role in achieving jurisdictional control. Knowledge can take on multiple forms, ranging from indeterminate to technical (I/T ratio) and from everyday to exclusive knowledge. To investigate the interrelatedness of jurisdiction, knowledge claims and professional status, we examine which knowledge claims were made by the medical and nursing professions in the Netherlands to secure or obtain, respectively, jurisdictional control over prescribing, and which form this knowledge took. The study is based on thirteen semi-structured stakeholder interviews and an extensive document analysis. We found that the nursing profession in its knowledge claims strongly emphasized the technicality and everyday knowledge character of the prescribing task, by asserting that nurses were already prescribing medicines, albeit on an illegal basis. Their second claim focused on the indeterminate knowledge skills of nurses and stated that nurse prescribing would do justice to nurses’ skills and expertise. This is a strong claim in a quest for (higher) professional status. Results showed that the medical profession initially proclaimed that prescribing should be reserved for doctors as it is a task requiring medical knowledge, i.e. indeterminate knowledge. Gradually, however, the medical profession adjusted its claims and tried to reduce nurse prescribing to a task almost exclusively based on technicality knowledge, among others by stating that nurses could prescribe in routine cases, which would generate little professional status. By investigating the form that professional knowledge claims took, this study was able to show the interconnectedness of jurisdictional control, knowledge claims and professional status. Knowledge claims are not mere rhetoric, but actively influence the everyday realities of professional status, interprofessional competition and jurisdictional division between professions.

## Introduction

Over the past decades, professional boundaries in health care have come under pressure, among others as a result of flexible neo-liberal approaches to managing health care [1,2]. The number of countries where nurses are legally permitted to prescribe medication has grown considerably [3-5]. Recently, the creation, establishment and renegotiation of boundaries has become a key theme in the sociology of professions [6]. In this article we are concerned with the negotiating of professional boundaries by the nursing and medical professions when it comes to the task of prescribing medicines.

Because prescribing has traditionally been the sole domain of the medical profession [7-9], the expansion of prescriptive authority to include nurses touches on issues of professional domains and competition between professions for jurisdiction over tasks. Jurisdiction or control over certain task areas is crucial for professions, because it is their means of continued livelihood [10]. Professionals who are recognized as experts in a certain area, in this case the area of prescribing medicines, typically possess a form of cultural capital whose ownership confers status and power [11]. Moreover, these professions often enjoy a number of privileges, such as control over professional training, recruiting and licensing [12]. Apart from the direct benefits, these help them to sustain their position in competition with other professions. Therefore, Abbott [12] labels jurisdiction – “the link between a profession and its work” (page 20) – as the central phenomenon of professional life.

Within jurisdictional domains, professions tend to make more or less exclusive claims to authority over the knowledge and skills that fall within their scope [13]. Knowledge claims play an important role in achieving jurisdictional control [11,14]. In this article, we adopt a broad understanding of knowledge claims as claims to unique bodies of knowledge and/or expertise. Because one profession can pre-empt another’s jurisdiction or control over a task, professions exist in an interdependent system with competing jurisdictional claims [12]. Consequently, when one profession aims to achieve more jurisdictional control in a certain task area, in this case the prescribing of medicines, other professional domain boundaries are inevitably affected as well [1].

In general, the relationship between the medical and nursing professions is referred to as the classical case of a dominant profession controlling a subordinate profession [12,15,16], even though it has been shown that on the work floor role blurring and informal crossing of boundaries takes place between doctors and nurses [17,18]. Nonetheless, the medical profession seeks to maintain its dominant position in the provision of health care [14,19] whereas the nursing profession tries to increase its professional status. Porter [20] and Gerrish et al. [21] describe several strategies of occupational advancement used by nurses over the last years, such as managerialism and the introduction of Master level nurse education, both aimed at expanding nursing’s scope of practice. The introduction of nurse prescribing can be viewed as a new chapter in the ongoing process of boundary negotiations between the medical and nursing professions. This is especially salient as prescriptive authority is seen by both professions as an important asset in maintaining and/or enhancing professional status [22].

In this article, we describe the introduction of nurse prescribing in the Netherlands from a sociology of professions perspective. Given the significance of jurisdiction in professional life, we focus on the knowledge claims made by the medical and nursing professions to secure or obtain, respectively, jurisdictional control over prescribing and related professional status. We examine what form these knowledge claims took and how they relate to the professional status of the professions involved. After all, knowledge claims are not mere rhetoric. They influence the everyday realities of professional status, interprofessional competition and jurisdictional division between professions.

### Professions, Knowledge Claims and Jurisdictional Control

Even though much research has focused on professions, no comprehensive and generally accepted definition of the concept “profession” has been developed. We define professions as “exclusive occupational groups applying somewhat abstract knowledge to particular cases” (page 8) [12]. From this definition, it follows that knowledge and its degree of abstraction are important currencies of competition between professions. This is reflected in the strategies used by professionals to secure or obtain professional or expert status and jurisdictional control. As McLaughlin and Webster [11] state, professional knowledge claims play an important role in achieving jurisdictional control and expert or professional status, and they represent an important vehicle through which professions can rhetorically play out their professional struggles [14].

As said before, we examine the knowledge claims put forward by the medical and nursing professions in their struggle for authority over prescribing. These knowledge claims are not made in a vacuum [23]. Professions exist within a wider social structure in which for example the government creates the legislative framework in which knowledge claims can be made. Naturally, professions will (implicitly) adjust their claims with reference to this legislative framework. However, our focus is on the knowledge claims themselves and how they relate to the professional status of the professions involved. This means that we discuss the role of the state only where it actively influences the knowledge claims that were used. Moreover, we do not comment on the success of these claims in terms of some measurable outcome.

Professional competition over jurisdiction can have various outcomes [12]. After all, not every profession striving for full jurisdiction will obtain it. Most professional conflicts over jurisdiction result in so-called “limited jurisdictional settlements” (page 71) [12]. These are alternatives to the situation in which one or more professions hold full jurisdiction over a task. In a jurisdictional settlement, professions share the jurisdiction over a task, whereby control is to a greater or lesser extent equally distributed between the professions, depending on the type of jurisdictional settlement concerned. Abbott [12] discerns several jurisdictional settlements, including: *subordination*, whereby an incumbent profession controls the division of labor for one or more subordinate groups, and *intellectual jurisdiction*, in which the incumbent profession controls the cognitive knowledge of an area but allows practice by other professions. It is possible that in the course of a professional conflict, professions adjust the jurisdictional goal they are striving for, such as when professions believe that the goal of full jurisdiction is no longer attainable. This might be reflected in the knowledge claims they are using. The state is an important influencing factor in this regard, because it can change the laws and regulations under which professions develop and use their knowledge claims.

Although Abbott [12] in his definition of professions states that abstract knowledge is important for professional status, he does not say much about the form of knowledge. Professional knowledge, however, can take on multiple forms. The form it takes influences the strength of jurisdictional claims. Jamous and Peloille [24] introduced the indetermination/technicality ratio (I/T ratio) to conceptualize the notion of professional knowledge form, enabling knowledge to be placed along a continuum from highly technical to highly indeterminate. The I/T ratio focuses on the transmissibility of knowledge, i.e., the part played in a production process by “means” that can be mastered and communicated in the form of rules (T), in proportion to the “means” that escape rules and are attributed to virtualities of producers (I) [24]. Hence, technicality refers to knowledge which can be codified, broken down into constituent tasks, rationalized and delegated. Think for example of the task of prescribing medicines based on medical guidelines and protocols. Indetermination is described as a skill associated with professional judgment, i.e., tacit knowledge, based on authority that is “acquired” through experience, ascription or initiation [11,14,24]. For example, prescribing medicines for frail elderly with multiple morbidity falls into this category.

A second distinction that is often made is between “exclusive” knowledge and “everyday” knowledge. Following Hirschkorn [14], we define exclusive knowledge as knowledge that is monopolized by and exclusively used by a particular professional group, whereas everyday knowledge is accessible to an undefined number of occupational groups and even to the lay public. This leaves us with a broad knowledge field, in which professional knowledge forms can be situated relative to their indeterminacy/technicality as well as relative to their level of exclusivity.

**Figure 1:**
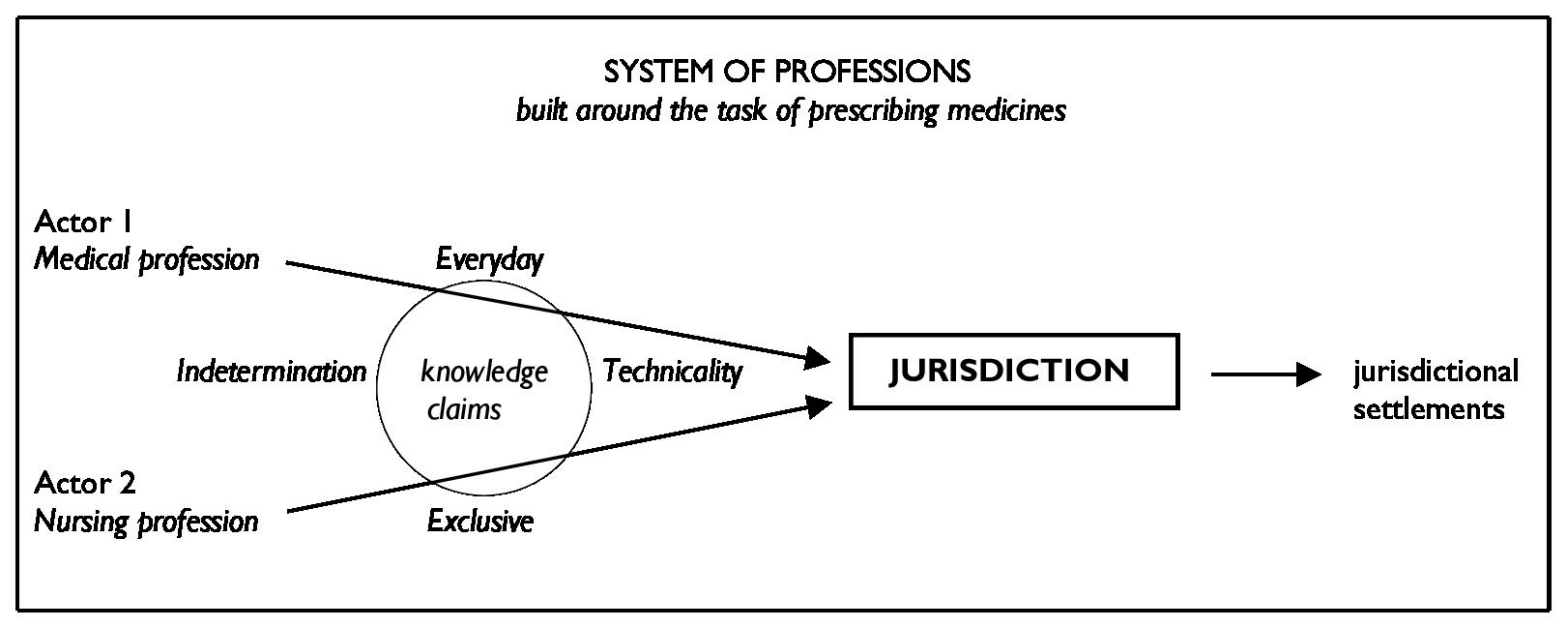
Graphic and partial representation of interprofessional conflict over prescribing.

Figure 1 shows a partial graphic representation of interprofessional conflict over the task of prescribing medicines. It depicts the relationships between professions, their knowledge claims and jurisdiction. It should be emphasized that this is a partial representation, because the system of professions exists within a wider social structure.

When it comes to professional struggles for jurisdictional control, medicine and nursing are facing a dilemma as to finding a balance between technical and indeterminate knowledge claims and everyday and exclusive knowledge claims. If they account for their knowledge and subsequent practice too strictly in terms of technical complexity and rules (e.g., medical guidelines and protocols), they risk the possibility of being taken over by other professions [12,13,25]. On the other hand, if they claim that certain knowledge is indeterminate, meaning that only their profession is gifted with that particular knowledge, the door is by definition closed for others to claim that knowledge as well. However, too much emphasis on indeterminacy is also dangerous. After all, other occupational groups can claim equal or superior indeterminate skills over the task at stake. Moreover, knowledge claims that suffer from too high a level of indeterminacy, will fail to convince the audience of their legitimacy. The most effective professional claims therefore seem to consist of both technical and indeterminate knowledge [13,25]. It is also important for professions to frame their knowledge as exclusive knowledge. After all, everyday knowledge, i.e., knowledge that is accessible to many occupational groups and sometimes even to the lay public, can by definition not be claimed. Moreover, everyday knowledge is not beneficial toward enhancing professional status. Therefore, successful professional claims usually emphasize the exclusive character of the knowledge that they possess.

### The Context of Nurse Prescribing

Nurse prescribing in the Netherlands is regulated by two different articles of law, one for registered nurses and one for nurse specialists (Master’s in Advanced Nursing Practice). At the time of writing, registered nurses are not yet allowed to prescribe medicines. Their prescriptive authority is regulated in article 36 of the Individual Health Care Professions Act, which states that prescriptive authority can only be granted to specific categories of registered nurses (Bachelor’s degree) that are designated by a Ministerial Order. The categories of registered nurses that were initially designated to prescribe are diabetes care nurses, lung nurses and oncology nurses [26]. However, issues around the recognition of education are not fully secured yet. It is expected that diabetes care- and lung nurses will start prescribing in the course of 2013 and oncology nurses by January 1, 2014 [27]. They will be allowed to prescribe a limited number of medicines within set protocols and standards, after a diagnosis has been made by a doctor [26,28,29].

The legislation for nurse specialists came into force on January 1, 2012. Nurse specialists with a Master’s degree in Advanced Nursing Practice have broader prescriptive authority than diabetes care nurses, lung nurses and oncology nurses will get, and their authority is related to their area of expertise (i.e., acute care, chronic care, intensive care, preventive care or mental health care). However, their prescriptive authority is part of the so-called experimental article (36A) in the Individual Health Care Professions Act. This means that nurse specialists are allowed to perform reserved procedures, including the prescribing of medicines, for an experimental period of 5 years. After a positive evaluation, a final settlement might be included in the law which will grant nurse specialists final authority to perform reserved procedures, including prescribing [30-32].

In the Netherlands, the prescribing of medicines was traditionally the exclusive domain of doctors. But since the beginning of this century, several reports have appeared that promoted task substitution in health care [33,34]. When nurse prescribing was for the first time discussed, several possible barriers to task substitution were reported, of which professional domain thinking was considered “the most persistent problem” (page 37) [34]. Hence, nurse prescribing is a development in which professional boundaries are disputed and jurisdictional control is at stake. In the years prior to the introduction of nurse prescribing, the medical and nursing professions actively tried to influence the public and policy debate. In this study, we examine the knowledge claims used by the medical and nursing professions to secure or obtain, respectively, jurisdictional control over the task of prescribing medicines.

## Methods

Data were collected using a multi-method approach consisting of semi-structured interviews with stakeholders on nurse prescribing in the Netherlands and an extensive document analysis. Stakeholder interviews provided the primary source of data. We aimed to include representatives of all organizations that were involved in the nurse prescribing debate in the Netherlands. This included representatives of overarching nurses and medical associations as well as more specialist associations, such as the Association for Diabetes Care Professionals (EADV) and the Dutch College of General Practitioners (NHG). A list of key organizations was compiled in consultation with experts on nurse prescribing from the Royal Dutch Medical Association (KNMG) and the Dutch Nurses’ Association (V & VN). Potential informants were also selected in consultation with these experts and were approached by the researchers to take part in the study. Representatives received an information letter explaining the aims of the study, the voluntary nature of participation and an invitation to participate in an interview. Participant consent was assumed upon accepting this invitation and participation in an interview.

Of the 16 representatives invited per email and telephone, 13 ultimately participated (see Table 1 for a list of all interviewed stakeholders). Twelve interviews were with a single informant and one was with two representatives of one organization interviewed together. The informants held policy- or board positions within their organization, and their answers represent the organization’s point of view. Every interview was conducted by one or two researchers (MK, LVD, PG and/or AF) who were trained in qualitative interviewing techniques. The interviews were semi-structured and were guided by a topic list that was drafted after the findings of an earlier systematic review of the literature on nurse prescribing [35]. Interview topics were: general information about the informant/organization, vision on nurse prescribing, degree of support for nurse prescribing, introduction of nurse prescribing, the legal-, educational- and organizational conditions for nurse prescribing, and challenges and threats to the work of doctors and nurses because of nurse prescribing. All interviews but one were recorded and a summary of the interview was sent to each representative to be edited, where necessary, as an accurate representation of the organization’s viewpoint. Representatives could mark sections of the interview summary as ‘off the record’, in addition to sections they already noted as ‘off the record’ during the interview itself. All ‘off-the-record’ requests (n=2) were granted in full. One interview was conducted by letter, at the request of the organization. The approved interview summaries formed the basis for analysis.

**Table 1:** List of interviewed stakeholders.

*Nursing associations*
Dutch Nurses’ Association (V&VN)
Association for Diabetes Care Professionals (EADV)
Association of Nurse Specialists (V&VN VS/NP)
Association of Lung and Oncology Nurses (V&VN L/O)
*Medical associations*
Royal Dutch Medical Association (KNMG)
National Association of General Practitioners (LHV)- *written*
Dutch College of General Practitioners (NHG)
Netherlands Association of Internal Medicine (NIV)- *telephone interview*
Dutch Association of Elderly Care Specialists (Verenso)
*Other stakeholders*
Health Care Inspectorate (IGZ)
Royal Dutch Pharmacists Association (KNMP)
Ministry of Health, Welfare and Sport (VWS)
Dutch Patients and Consumers Federation (NPCF)

No ethical approval was deemed necessary for this study as the information that was collected did not refer to peoples’ individual opinions or behaviors but exclusively to organizational points of view concerning nurse prescribing. However, all informants consented that the approved interview summaries, in which their organizations were mentioned by name, could be used in research publications. Moreover, all informants were informed that they could withdraw from the study at any time during or after the interview. All data collected were handled as required by the rules of the Dutch Data Protection Act (Dutch: Wbp- Wet bescherming persoonsgegevens) and the applicable codes of conduct for scientific researchers. Raw data (i.e. the approved interview summaries) are available upon request from the first author, but only after permission from the organization concerned has been obtained.

In addition to stakeholder interviews, document analysis provided information that was used to supplement data collected through interviews. Considerable effort was made to obtain relevant documents, such as policy documents, position papers, newspaper articles, letters to the Minister of Health, and government documents regarding nurse prescribing, from various sources. These sources included the websites of the associations that were interviewed, digital archives of their professional journals, digital government archives and the LexisNexis database of national newspaper articles. Because most of these websites lacked advanced search facilities, we used combinations of the following keywords, where possible, to search for relevant documents from the last 10 years: “prescriptive authority”, “nurses”, “nurse specialists”, “prescribing”, “medicines” and “task substitution”. For government archives, the additional search terms “32.196” and “32.361” were used, because these were the numbers of the (draft) bills on prescriptive authority for nurses. Documents selected for inclusion were searched manually to identify further relevant documents. We included all documents in which knowledge claims were expressed by either (a representative of) the medical profession, the nursing profession, or both; where these knowledge claims referred to (the introduction of) nurse prescribing; and where there was no question of individual views. We included a total of 34 documents in the study. The oldest document included dates back to 2003, but the majority of retrieved documents was from recent years.

We performed a thematic analysis of the approved interview summaries and documents gathered through the document analysis [36]. Data analysis began at an early stage in the research to introduce any necessary changes in the interview protocol. Data were coded using MAXQDA 2007 qualitative data analysis software [37] and were analyzed both inductively and deductively. Guided by our theoretical model, we searched the data for concepts that were directly linked to interprofessional tensions around nurse prescribing. Additionally, data were analyzed inductively and compared for common statements and claims. Subsequently, recurring themes were identified and classified, and text fragments were sorted according to the thematic framework. Three of the researchers took part in internal discussions of the analysis and themes were discussed until consensus was reached. Analysis of the data identified the following thematic elements: illegal nurse prescribing, professional domains, (exclusive) task/knowledge area doctor/nurse, preconditions for nurse prescribing, protocols/guidelines, comorbidity/polypharmacy and routine aspects of prescribing. Based on these themes, we distinguished the knowledge claims used by the nursing and medical professions. Quotations were chosen to illustrate the knowledge claims. It should be noted that these quotations came from the interview summaries that were approved by the interviewees.

Our study has largely been reported according to the COREQ guidelines [38], see Checklist S1.

## Results

### Knowledge Claims by the Nursing Profession

The main argument of the nursing profession in seeking prescriptive authority was that nurses were already prescribing medicines, albeit on an illegal basis. This claim was repeatedly cited by all nursing organizations that were involved in seeking prescribing rights, implying that it would only be logical to grant nurses legal prescribing rights as well. After all, nurses had proven to be competent to prescribe. The Dutch Nurses’ Association (V & VN) put it like this in their interview with us:

*The pragmatic question for prescribing rights came from the nursing profession itself. From the field, more and more signs emerged that certain groups of nurses, although unauthorised, nonetheless often prescribed medicines*.

The newsletter from the Association for Diabetes Care Professionals (EADV) of March 2007 was also explicit in this regard:

*V&VN has been pleading for a long time already to formalize nurses’ position in the administration of drugs. For years, nurses have been prescribing medicines without having the competence to do so [39].*

Moreover, in our interview with a representative of the Dutch association for lung nurses (V & VN Longverpleegkundigen) it was stated that prescribing by nurses was “a daily practice”.

The fact that nurses were already prescribing medicines, despite the lack of a legal framework, had long been openly acknowledged by all parties involved. Even the Royal Dutch Medical Association (KNMG) acknowledged this in their interview with us by mentioning that in practice, diabetes care nurses, lung nurses and oncology nurses “already prescribe together with the relevant doctor”. However, once the idea of legal nurse prescribing was mooted, these existing prescribing practices became an important factor for the nursing profession to plead for official prescribing rights. The profession wanted recognition for the work nurses had already been doing for years. They wanted to be recognized as prescribers.

By repeatedly referring to the fact that nurses were already prescribing medicines in daily practice, however, the nursing profession (unintentionally) emphasized the everyday knowledge character of prescribing, or at least the everyday knowledge character of that part of the prescribing task for which they were claiming jurisdiction. After all, nurses were not prescribing all medicines, they had only “learned” part of the prescribing job. They were now claiming legal jurisdiction over precisely that part of the prescribing task that they had themselves shown to be susceptible to incursion. Although this can be a pragmatic claim for obtaining legal prescribing rights, it is a much weaker argument in nurses’ search for (more) professional status, because it strongly emphasizes the everyday knowledge character of the task and the technicality side of the I/T ratio.

A second related claim that was constructed and repeatedly put forward by the nursing profession to acquire legal prescribing rights was that the introduction of nurse prescribing would do justice to nurses’ skills and expertise. Sometimes, it was even claimed that nurses were better at prescribing than doctors, because nurses had a better view of patients and could “see how someone stands in life”. The president of the Dutch Nurses’ Association (V & VN) repeatedly summarized the “crucial role” that nurses played in the administering and prescribing of medicines, stating that a nurse:

*(..*)*has good contact with him [the patient*]*, observes him well, writes a prescription face to face, provides information, can immediately answer questions and can monitor the use, effects and side-effects of the medicine. Nowadays, these things do not happen enough, the doctor has too little time to do it [40].*

Moreover, in an open letter to a major Dutch newspaper (NRC Handelsblad), the president of the Royal Dutch Medical Association and the president of the Dutch Nurses’ Association in 2010 jointly wrote that:

*Many tasks in health care can be performed better by nurses and nurse practitioners than by doctors [41].*

The president of the Association for Diabetes Care Professionals (EADV) in her interview likewise claimed that “the diabetes care nurse is thé expert in the field of adjusting and regulating insulin”. So, besides pointing out that nurses were already prescribing medicines, the nursing profession explicitly represented nurses as “the experts” in prescribing medicines. The profession underpinned this claim to exclusive knowledge by stating that nurses were providing doctors with medication advice. Moreover, the profession argued that nurses believed they had a better understanding of patients than doctors. This is evident in the following quote from our interview with the Dutch Nurses’ Association (V & VN):

Moreover, it came to the fore that nurses had the idea that they had a better view on patients than the doctor or general practitioner, because they have a much broader view and, for example, can see how someone stands in life.

Because these claims hinge on the exclusive talents of nurses, they emphasize the indeterminate character of nursing knowledge. Hence, this is a stronger claim in nurses’ quest for (higher) professional status, because it emphasizes the exclusive talents of nurses.

### Knowledge Claims by the Medical Profession

When nurse prescribing was first discussed in the Netherlands as a realistic possibility in health care, the medical profession was outspoken in opposing the proposal. The medical profession proclaimed that the prescribing of medicines should be “reserved to doctors” [42], among others because it feared prescribing errors and the loss of coherence in patients medication policy.

Initially, the medical profession’s main angle of resistance focused on prescriptive authority for registered nurses, i.e., diabetes care nurses, lung nurses and oncology nurses. The medical profession emphasized that these categories of nurses were not legally identifiable, because their specialization (diabetes, lung and oncology care) cannot be laid down in law, because the law only contains the category “registered nurse”. Therefore, it would likewise be impossible to identify these groups of nurses as legal prescribers, and accordingly they should not be granted prescribing rights. Furthermore, the medical profession was concerned about their lack of diagnostic skills and knowledge of comorbidity and polypharmacy. According to the medical profession, “only a doctor is capable of diagnosing” (page 8) [43] whereas nurses lack the broad integral knowledge and skills to take comorbidity and polypharmacy into account. Hence, the medical profession emphasized the indeterminate character of the knowledge, i.e., medical knowledge, required for prescribing. The following illustrative quote is from an interview with the Dutch National Association of General Practitioners (LHV):

*When prescribing medicines, interactions with other medicines may develop. The specialized nurse lacks the polypharmaceutical knowledge that is needed to oversee complications caused by polypharmacy.*

Gradually, however, a change in claims can be discerned. In 2006 for example, the title of a news article on the website of the umbrella medical organization (KNMG) read “Nurse prescribing finds favor in the eyes of the KNMG” [44]. Even though this heading revealed an authoritative stance, it also showed, albeit unwillingly, a slightly more positive outlook on nurse prescribing. Moreover, it should be noted that within the medical profession, there was less resistance against prescriptive authority for nurse specialists (Master’s in Advanced Nursing Practice), with the exception of the general practitioner associations, who claimed, among other things, that the proposed legislation for nurse specialists contained too little conditionality to guarantee the safety of prescribing. The Dutch College of General Practitioners (NHG) mentioned in their interview with us that because of the legislation:

*The need for consultation [between a doctor and nurse specialist*]* falls away and cooperation agreements lose their obviousness.*

Over time, part of the medical profession altered its claims and started to claim that a small part of the prescribing task could be done by nurses as well. Where “routine tasks” and prescribing based on measured values were concerned, and where cooperation with a doctor would be guaranteed, the medical profession believed that prescribing by nurses could be feasible, albeit for a limited number of medicines. In 2010 the Dutch National Association of General Practitioners (LHV), for example, stated that prescribing by diabetes care nurses and lung nurses would not be a problem, because they would “only prescribe on the basis of measured results” (page 8) [43]. The quote below from our interview with the Dutch Association of Elderly Care Physicians (Verenso) also describes this stand:

*Regarding the prescriptive authority for nurse specialists, Verenso is of the opinion that nurses should prescribe by treatment protocols in which medication quantities et cetera should be specified.*

The following quote from an article by the Royal Dutch Medical Association (KNMG) from 2011, relating to nurse specialists, likewise reflects the tentative nature of the medical profession’s agreement with task substitution to nurses and especially nurse prescribing:

*The KNMG also thinks that in the additional rules [to the law*]* at least the following should be regulated to ensure the quality of care: national guidelines for indicating and performing certain medical procedures, cooperative arrangements between the relevant professionals and doctors and the condition that task substitution takes place only for routine tasks for which the risks are sufficient to grasp [30].*

It is clear that the medical profession gradually became less negative about nurse prescribing and started to see some room for (limited) nurse prescribing right. However, it should be noted that the part of the prescribing task that the medical profession was willing to share and/or hand over to nurses, was reduced to a task almost exclusively built on technical (T) knowledge. After all, prescribing based on measured values, guidelines and protocols is characterized by a high level of codified knowledge that can be mastered and communicated in the form of strict rules.

From the interviews and document analysis, it seems that the medical profession quite early on in the process believed it would be wiser to put its energy into arranging nurse prescribing in such a way that the outcomes would be as beneficial as possible for itself, instead of continuing to resist it. The Royal Dutch Medical Association (KNMG), for example, mentioned in their interview with us that:

*One of the conditions that the KNMG would then have liked to include in the law, but for which she was unable to raise sufficient support in the House of Representatives, was that nurse specialists would be required to prescribe within a mandatory partnership, including at least one physician.*

And in 2006 already, a negative KNMG comment about how task substitution was legally regulated, was followed by the sentence

*Anyhow, it now comes down to the point that the conditions under which [nurse*]* prescribing can take place, are in place [45].*

Throughout the years, the medical profession repeatedly made this kind of fatalistic comments, almost always followed by statements underlining the importance of a proper arrangement of the conditions under which nurse prescribing should be introduced.

Increasingly, the medical profession emphasized that nurse prescribing should be based on protocols and guidelines that should be developed by the professional groups, i.e., registered nurses and nurse specialists, and doctors together, again stressing the value they placed on technical knowledge. In an open letter to the Chairperson of the Dutch House of Representatives the Royal Dutch Medical Association (KNMG) in 2011, for example, wrote that prescribing should be performed using “written cooperative arrangements between the professionals involved in the task reallocation” (page 3) [46], and the Dutch National Association of General Practitioners (LHV) claimed that specific protocols should be drafted by “the concerned professional groups” (page 8) [43]. By focusing on the medical profession’s crucial role in the drafting of new protocols and guidelines for nurse prescribers, the profession tried to retain intellectual jurisdiction over prescribing.

## Discussion

In the debate on nurse prescribing in the Netherlands, both the nursing and medical professions used various knowledge claims to obtain or secure, respectively, jurisdictional control over prescribing. These knowledge claims were closely connected with their professional boundaries, professional status and the kind of jurisdictional control they were aiming for.

The claim of the nursing profession that nurses were already prescribing medicines, albeit on an illegal basis, was pragmatic in terms of obtaining legal prescribing rights and the expansion of nurses’ professional boundaries, but less effective for enhancing their professional status. After all, it showed that the particular part of the prescribing task that nurses were claiming jurisdiction over, was built up of technical knowledge that could easily be taken over by other professionals. The other main knowledge claim of the nursing profession – that nurses were thé experts on prescribing – might have been less pragmatic in terms of actually expanding the boundaries of the nursing profession, because it is a claim that is difficult to demonstrably substantiate, but it was more appropriate in aiming for professional status enhancement, because professionals who are recognized as experts in a certain area typically possess status and power [11].

The medical profession initially insisted that nurses should not be granted prescribing rights, because one needs a broad medical vision to prescribe. By focusing on the indeterminate character of prescribing knowledge, the medical profession stubbornly tried to defend its professional boundaries and keep full jurisdiction over prescribing of medicines. However, in the course of the debate, the claims used by the medical profession changed and appear to have been aimed toward other jurisdictional goals. The medical profession started to see room for limited nurse prescribing rights and started to emphasize the technical and routine character of the prescribing tasks that nurses could perform. This professional strategy, in which nurses’ work is denoted as “routine”, is not uncommon. Sanders and Harrison, for example, showed that both geriatricians and GPs employed a discourse that strongly emphasized the routine elements of specialist heart failure nursing work. By contrasting their own work with the routine tasks performed by these nurses, geriatricians and GPs tried to emphasize the autonomy of their own role [2].

Hence, the medical profession gradually allowed a shift in its own professional boundaries, by allowing nurses to prescribe as well. However, at the same time the profession tried to secure its own professional status and minimize the enhancement of nurses’ professional status. After all, routine tasks are a target for deprofessionalization, as Abbott [12] states, and by delegating the “dangerous” routine part of the prescribing task to nurses, the result might be “the degradation of what had been professional work to nonprofessional status” (page 126) [12]. Additionally, by claiming that nurses should only prescribe via guidelines and protocols that were developed in collaboration with doctors, the medical profession skillfully defended its own professional status by aiming for intellectual jurisdiction over prescribing.

The fact that the medical profession gradually changed its knowledge claims and its jurisdictional aims, is not unique for a debate in the Netherlands, as the American historian Kennedy [47] showed. In his analysis of the creation of Dutch euthanasia law, he showed that even prior to the introduction of euthanasia legislation, it was already openly stated that (illegal) euthanasia requests were sometimes granted. In this climate of open discussion, the eventual liberalization of euthanasia became an inevitable development in the eyes of many, and even critics and opponents believed they would do better to focus on an adequate regulation of this inevitable practice instead of continuing to resist it [47]. It is quite possible that the medical profession in the Netherlands believed the same when it saw itself confronted with the open discussion about nurses prescribing medicines, even though this was officially prohibited. Instead of resisting the introduction of nurse prescribing, the medical profession aimed for adequate regulation and tried to preserve its intellectual jurisdiction.

Moreover, it should be noted that professions, implicitly or explicitly, adjust their claims to the legal framework in which they are operating. In the Netherlands in recent decades, policy makers as well as successive governments adopted an increasingly favorable attitude to task substitution, whereas the legal possibilities for task substitution were extended. Together with the open discussion climate in the Netherlands, this might have contributed to the medical profession’s outlook on nurse prescribing as an inevitable development and might have influenced its knowledge claims.

Although we provide insight into how the form of knowledge claims can influence jurisdictional conflicts at the level of professional associations, we cannot make any statements about how these claims will affect the division of jurisdictional control on the work floor. As Abbott notes, the work floor is a separate jurisdictional arena, and claims made in the workplace often distort the official lines of legally and publicly established jurisdiction [2,12], as was for example shown by Allen [17] and Snelgrove and Hughes [18] in their studies on role blurring and informal boundary crossing between doctors and nurses. Nonetheless, considering that struggles take place on organizational level between the nursing and medical profession concerning prescribing, our study suggests that good communication will be an important factor in the successful introduction of nurse prescribing in practice. Moreover, we did not evaluate the knowledge claims used on their factual accuracy. We wanted to examine what medicine and nursing claimed as their knowledge and why. By the same reasoning, we did not comment on the success of these knowledge claims in terms of some measurable outcome. Whether knowledge claims were based on facts, to what extent they held true, and to what extent they were successful was irrelevant for this study, although these are interesting questions for further research.

Even though we studied knowledge claims used by two specific professions in their particular quest for jurisdictional control over prescribing of medicines, our study is of wider interest in the context of contemporary health care policy. Nurse prescribing has been introduced in eight Western European and Anglo-Saxon countries over the past two decades [35,48], resulting in increasing professional boundary negotiations between medical and nursing professions internationally. For example in Australia, Sweden and the USA, medical associations mainly opposed nurse prescribing and in Spain, which is currently in the process of introducing nurse prescribing, the General Council of Physicians is against granting nurses the legal authority to prescribe medicines [4,49-51]. Medical and nursing professions in these countries are competing with each other over the jurisdiction over prescribing and in the process likewise make use of knowledge claims.

Moreover, the prescribing of medicines is by no means the only task substitution that is taking place. Task substitution is increasingly seen as a solution to current problems in health care, for example in the Netherlands [33,34] but also internationally [2,52,53]. In the light of these developments, professional boundaries are and will be increasingly contested. As a result, professions will be forced to develop knowledge claims to defend their established jurisdictions, obtain new jurisdictions and redefine their professional status. Because after all, jurisdiction is the central phenomenon of professional life [12].

## Supporting Information

Checklist S1 **COREQ checklist Kroezen et al. (2013).**
(DOCX)
