# Supplementary material for: Knowledge Claims, Jurisdictional Control and Professional Status: The Case of Nurse Prescribing
Source: PLoS One. 2013 Oct 4;8(10):e77279. doi: 10.1371/journal.pone.0077279 (PMC3790745; doi:10.1371/journal.pone.0077279)
Supplement: Checklist S1 — COREQ checklist Kroezen et al. (2013). (DOCX) [file pone.0077279.s001.docx]

**Checklist S1. Completed COREQ checklist [38] Kroezen et al.**

| **Domain 1: Research team and reflexivity** |
| --- |
| *Personal characteristics* |
| 1. It is reported that every interview was conducted by one or two researchers (MK, LvD, PG and/or AF) who were trained in qualitative interviewing techniques |
| 1. Credentials of all researchers are reported to PLOS ONE. Marieke Kroezen, BSc (Hons), MSc Liset van Dijk, PhD Prof. Peter Groenewegen, PhD Prof. Anneke Francke, PhD, RN |
| 1. The authors’ occupations are not reported. The first author is PhD researcher at NIVEL. The second author is Programme coordinator Pharmaceutical care at NIVEL. The third author is Director at NIVEL and Professor at Utrecht University. The fourth author is Programme coordinator Nursing care at NIVEL and Professor at VU University Amsterdam. |
| 1. It is reported (by first names) that the third author is male and that the first, third and fourth authors are females. |
| 1. It is reported that all authors have extensive training and experience in performing qualitative research (including conducting in-depth interviews). |
| *Relationship with participants* |
| 1. No relationship was established with informants prior to study commencement. |
| 1. It is reported that all participants were informed with an information letter about the purpose of the study. Additionally, the study was explained at the time of interview. |
| 1. Participants were informed that they were free in their answers and that there were no ‘good or wrong answers’. |
|  |
| **Domain 2: Study design** |
| *Theoretical framework* |
| 1. It is reported that our were analyzed with thematic analysis. |
| *Participant selection* |
| 1. It is reported that a list of key organizations was compiled in consultation with experts on nurse prescribing from the Royal Dutch Medical Association (KNMG) and the Dutch Nurses’ Association (V&VN). Potential informants were also selected in consultation with these experts and were approached by the researchers to take part in the study. |
| 1. It is reported that potential representatives received an information letter explaining the aims of the study and were subsequently invited by email and/or telephone to participate in the study. |
| 1. It is reported that the sample consists of 13 representatives. |
| 1. It is reported that of the 16 representatives invited, 3 representatives did not participate. |
| *Setting* |
| 1. Data were almost always collected at the offices of the respondents. |
| 1. At interviews, no one else except the interviewee(s) and interviewer(s) were present. |
| 1. All important characteristics of the sample are reported. |
| *Data collection* |
| 1. Development of the questionnaire is discussed. |
| 1. It is reported that participants were interviewed once. |
| 1. It is reported that the interviews were audio-taped. |
| 1. Field notes were not taken as the interviews were audio-taped. |
| 1. Interview times are not discussed. |
| 1. Data saturation is not discussed. |
| 1. It is reported that a summary of the interview was sent to each representative to be edited, where necessary, as an accurate representation of the organization’s viewpoint. The approved interview summaries formed the basis for analysis. |
| **Domain 3: Analysis and findings** |
| *Data analysis* |
| 1. It is reported that three of the researchers took part in internal discussions of the analysis (MK, LvD, AF). |
| 1. No coding tree is reported. |
| 1. It is reported that the themes were derived both inductively and deductively. |
| 1. It is reported that data were coded using MAXQDA 2007 qualitative data analysis software. |
| 1. Participants did not provide feedback on findings. This is not mentioned. |
| *Reporting* |
| 1. Quotations were chosen to illustrate the knowledge claims. It should be noted that these quotations came from the interview summaries that were approved by the interviewees. For each quotation, the source is reported. |
| 1. There is consistency between the data presented and the findings. |
| 1. The results section is structured according to the major themes found in the data. |
| 1. There is no description of diverse cases and minor discussion of minor themes. |
